# Current state and future prospects of spatial biology in colorectal cancer

**DOI:** 10.3389/fonc.2024.1513821

**Published:** 2024-12-03

**Authors:** Francisco G. Carranza, Fernando C. Diaz, Maria Ninova, Enrique Velazquez-Villarreal

**Affiliations:** ^1^ Department of Integrative Translational Sciences, City of Hope, Beckman Research Institute, Duarte, CA, United States; ^2^ Lineberger Comprehensive Cancer Center, University of North Carolina, Chapel Hill, NC, United States; ^3^ Department of Biochemistry, University of California, Riverside, Riverside, CA, United States; ^4^ City of Hope Comprehensive Cancer Center, Duarte, CA, United States

**Keywords:** colorectal cancer, spatial transcriptomics, spatial proteomics, bioinformatics, genomics, personalized medicine, translational research, spatial biology

## Abstract

Over the past century, colorectal cancer (CRC) has become one of the most devastating cancers impacting the human population. To gain a deeper understanding of the molecular mechanisms driving this solid tumor, researchers have increasingly turned their attention to the tumor microenvironment (TME). Spatial transcriptomics and proteomics have emerged as a particularly powerful technology for deciphering the complexity of CRC tumors, given that the TME and its spatial organization are critical determinants of disease progression and treatment response. Spatial transcriptomics enables high-resolution mapping of the whole transcriptome. While spatial proteomics maps protein expression and function across tissue sections. Together, they provide a detailed view of the molecular landscape and cellular interactions within the TME. In this review, we delve into recent advances in spatial biology technologies applied to CRC research, highlighting both the methodologies and the challenges associated with their use, such as the substantial tissue heterogeneity characteristic of CRC. We also discuss the limitations of current approaches and the need for novel computational tools to manage and interpret these complex datasets. To conclude, we emphasize the importance of further developing and integrating spatial transcriptomics into CRC precision medicine strategies to enhance therapeutic targeting and improve patient outcomes.

## 1 Introduction: the role of spatial biology in colorectal cancer research

Colorectal cancer (CRC) is a multifaceted disease driven by the interplay of genetic, environmental, and lifestyle factors. Among these, age, family history, genetic syndromes, microbiota, and lifestyle choices such as diet and physical activity have been identified as significant contributors to its development and progression (1). CRC typically originates in the colon or rectum—parts of the large intestine—and commonly begins as benign growths known as polyps. Over time, these polyps can accumulate genetic mutations, transforming into cancerous and potentially metastatic forms (2). As cancer research has advanced into the precision medicine era, an emphasis on understanding molecular characteristics has emerged as a vital direction, particularly in CRC research. To uncover novel insights into cancer’s molecular underpinnings, researchers have increasingly turned to multi-omics approaches, including genomics, transcriptomics, proteomics, microbiomics, epigenomics, lipidomics, and pharmacogenomics (3). Beyond their use in basic cancer research, these multi-omics techniques are becoming pivotal in the diagnosis, prognosis, and personalized treatment of cancer patients, particularly for biomarker discovery across various cancer types (3–6).

The integration of multi-omics approaches has enabled a more comprehensive understanding of biological patterns, facilitating the development of more precisely tailored treatments and advancing the goals of precision medicine in cancer (7). This is particularly crucial for understanding CRC, a disease known for its heterogeneity within the tumor microenvironment (TME) (8, 9). The TME is a dynamic and intricate network of cellular and non-cellular components surrounding and interacting with cancer cells within a tumor. It significantly influences cancer development, tumor progression, invasion, metastasis, and treatment response (10–12). Research has demonstrated that tumor heterogeneity is a primary source of resistance to cancer therapies.

Additionally, CRC is the third most common cancer type worldwide, following lung and prostate/breast cancer, making it a major public health concern (13). CRC accounts for approximately 1.9 million cases and 0.9 million deaths annually, with numbers projected to double by 2040 (14). The economic burden is substantial, with $19 billion spent annually on healthcare and loss of work productivity in Europe, and $24.3 billion spent on healthcare alone in the U.S. in 2020 (14, 15). While the overall incidence of CRC has stabilized or decreased in higher-income countries, an alarming trend has emerged, showing an increase in CRC among younger populations. This trend has resulted in CRC becoming the leading cause of cancer deaths in men and the second in women under 50 years old (13, 16, 17).

This rise in early-onset CRC incidence and mortality has been attributed to factors such as increased sedentary lifestyles and the consumption of high-fat foods; however, the exact cause is likely multifactorial, involving both external and genetic health determinants (2, 17). Understanding the genetic and epigenetic alterations in each unique case of CRC is crucial in combating this disease. The advent of recent spatial biology technologies allow researchers to gain novel insights into the tumor microenvironment (TME) of CRC, potentially leading to applications in personalized medicine. In this review, we explore the current state and future applications of various spatial biology strategies in CRC research, addressing the key challenges associated with these approaches and discussing the significance of spatial transcriptomics and proteomics in tackling tumor heterogeneity in CRC. Additionally, we examine the integration of these strategies into precision medicine.

### 1.1 Spatial transcriptomics

The application of next-generation sequencing (NGS) techniques to CRC research has provided significant new insights into the genomic and epigenomic factors driving CRC development and progression (18, 19). These advancements have greatly enhanced our understanding of the molecular mechanisms underlying CRC, such as the transcriptome, leading to the identification of potential therapeutic targets and strategies for effective treatment (20, 21). NGS technology, along with the development of RNA-seq or bulk RNA-seq, has enabled researchers to ask whole-transcriptome questions and explore the molecular differences between healthy and cancerous cells while superficially examining the TME (18–20). Tumor heterogeneity is a key factor in tumor progression, metastasis, and treatment resistance (21). Bulk RNA-seq allows researchers to ask broad, lower-resolution questions about groups of cells or a tissue’s collective transcriptome, facilitating the categorization of tumors by molecular subtypes and providing rough estimates of different cell types. However, this broad overview of tissue transcriptomics has limitations in understanding the TME (22, 23).

One of the significant limitations of bulk RNA-seq is the loss of cell-type-specific and spatial context in understanding gene expression (24). To address these limitations, new technologies and methods to explore heterogeneity in the TME have emerged, such as single-cell RNA-seq (scRNA-seq) (25) and its variant single-nucleus RNA-seq. These methods rely on the physical separation of individual cells or nuclei and incorporating unique cell-specific barcodes to transcriptomic libraries. Thus, scRNA-seq technologies allow researchers to capture the transcriptome of individual cells, enabling the identification of rare cell types and the study of cellular heterogeneity within tumors at a higher resolution (23, 26). Despite its promise, scRNA-seq has its limitations, primarily because it requires tissue dissociation before library construction, resulting in the loss of spatial context. Spatial context provides vital information about each tumor’s unique microenvironment, cell-to-cell interactions, and organization, which are crucial for understanding the finer details of tumor biology.

In recent years, high-throughput spatial transcriptomics (ST) technology has emerged as an ideal approach to obtain the most comprehensive transcriptomic picture of a tumor’s microenvironment and elucidate CRC’s heterogeneity. Spatial transcriptomic methods address the shortcomings of bulk RNA-seq and scRNA-seq, offering a groundbreaking technology that allows cancer researchers to integrate NGS with spatial coordinates within a tumor. This technology combines tissue imaging, biomarker analysis, NGS, and bioinformatics to meticulously map gene expression across a tumor sample, providing unprecedented insights into tumor architecture and the interactions between different cell types. Thus, spatial transcriptomics offers a more complete understanding of the mechanisms behind cancer development and progression and presents new opportunities for CRC diagnosis and precision medicine.

Spatial transcriptomics comes in two main types: image-based methods, including *in situ* hybridization (ISH), *in situ* sequencing (ISS), and NGS sequence-based methods. Each category has multiple technologies, each with its own advantages and disadvantages for exploring the TME. While this technology is relatively new, it has already significantly impacted CRC research and is expected to continue to do so in the future.

## 2 Imaging-based spatially resolved transcriptomics in colorectal cancer research

**Figure 1:**
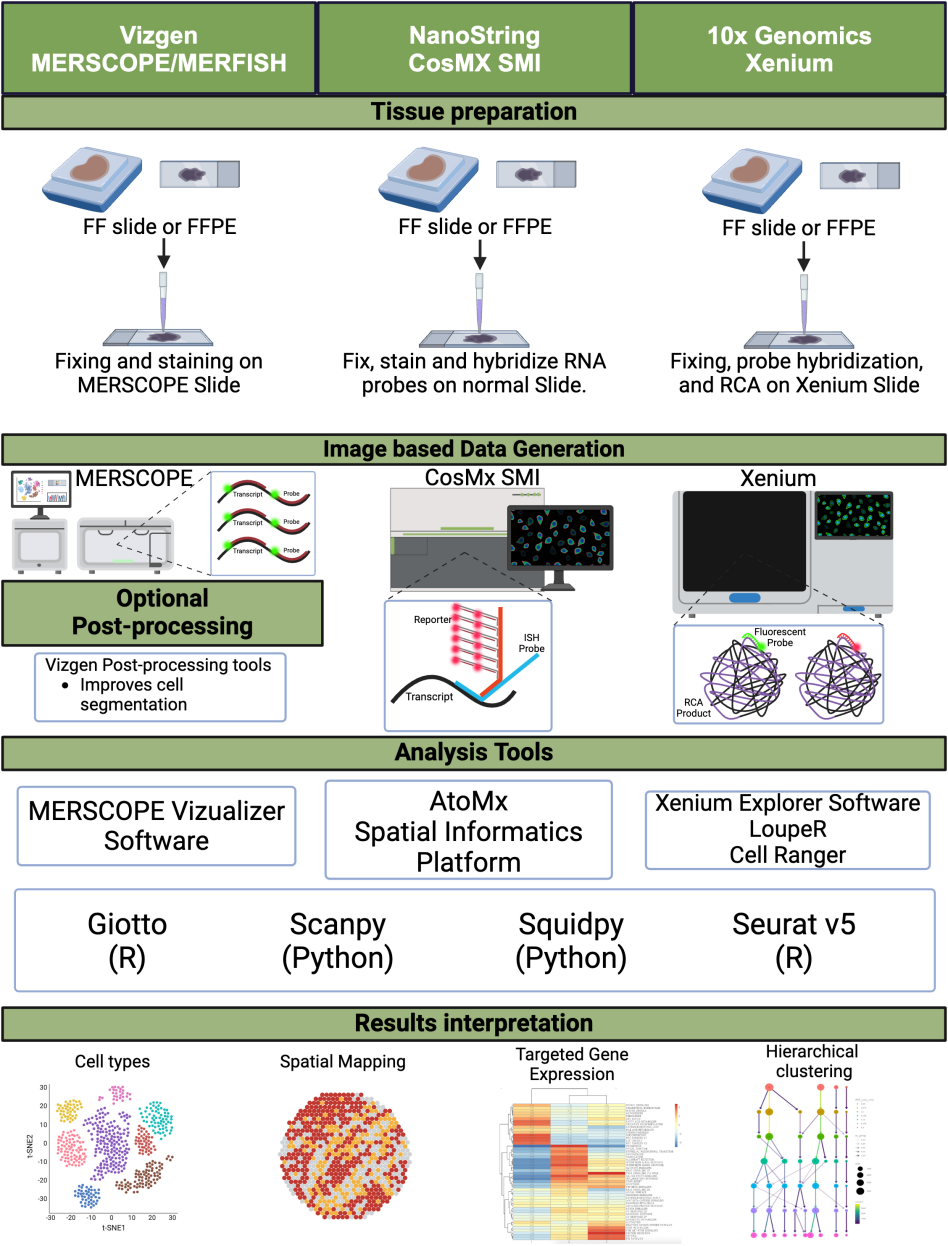
Image-based spatial transcriptomics. A schematic diagram depicting the workflow for image-based spatial transcriptomics platforms, from tissue preparation to data generation, including analysis tools and data interpretation. FF, fresh frozen; FFPE, formalin-fixed paraffin-embedded; RCA, rolling circle amplification. Created in BioRender. Carranza, F. (2024) BioRender.com/k99q194.

Imaging-based spatial transcriptomics (ST) techniques, including *in situ* hybridization (ISH) and *in situ* sequencing (ISS), have revolutionized the ability to map gene expression within the spatial context of tissue architecture. These methods utilize fluorescent probes or sequencing-based strategies to detect RNA molecules, preserving the tissue’s morphological backdrop and enabling high-resolution visualization of gene expression patterns. Unlike sequencing-based spatial methods, imaging-based ST approaches provide direct visualization of RNA transcripts, offering unique insights into the tumor microenvironment (TME) of CRC (**Figure 1**).

### 2.1 *In situ* hybridization platforms: MERFISH/MERSCOPE

The earliest applications of spatial transcriptomics in CRC research can be traced back to *in situ* hybridization (ISH) methods, which were initially used to map mRNAs while preserving spatial context (27). Over time, ISH evolved into single-molecule Fluorescent in Situ Hybridization (smFISH), a technique that allows the detection of single RNA molecules using multiple short probes that bind to different regions of the same RNA molecule. These short probes are fluorescently labeled and visualized with a fluorescence microscope, providing precise quantification of RNA molecules along with spatial context (28). The technology has significantly advanced with the introduction of high-throughput Multiplexed Error Robust Fluorescence in Situ Hybridization (MERFISH) in 2015.

Since 2021, Vizgen has commercialized this ST technology with their MERSCOPE and newly released MERSCOPE ULTRA platforms. MERFISH is an imaging-based ST method that enables single-cell imaging of the expression of up to 1,000 genes and thousands of RNA species through combinatorial FISH labeling, providing the ability to measure the copy number and spatial distribution of those RNA species. Despite the potential of this technology, it has not yet gained significant traction among CRC researchers. However, a notable study utilized Vizgen’s MERSCOPE public colon cancer dataset, comprising approximately 800,000 cells, to observe the expression pattern of secreted frizzled-related protein 2 (SFRP2) in fibroblast cells. This study identified SFRP2, a gene involved in the WNT signaling pathway, via scRNA-seq differential expression analysis. Through the use of Lactobacillus acidophilus postbiotics, they determined that they could target the differentially expressed genes to induce cell cycle arrest and promote anti-proliferative and anti-migration effects on a CRC cell line, thereby providing a new therapeutic avenue to explore (29).

### 2.2 NanoString CosMx spatial molecular imager

The CosMx Spatial Molecular Imager (SMI) is NanoString’s high-plex *in situ* platform for spatial multiomics, released in 2022. Similar to MERSCOPE, this platform leverages FISH-based probes that are designed to bind to specific RNA or protein targets, allowing for the capture of spatial context for approximately 6,000 genes and over 64 proteins at a single-cell and subcellular resolution on a tissue sample (30). CosMx SMI provides a unique platform to study RNA and protein expression simultaneously with spatial context. This capability allows researchers to integrate transcriptomics and proteomics data for a more comprehensive understanding of the molecular landscape. However, our extensive literature review found no peer-reviewed studies that have used CosMx SMI specifically in CRC research, suggesting that this technology is still in its early stages of adoption for CRC studies.

### 2.3 *In situ* sequencing platforms: 10x genomics xenium and cartana

In 2022, 10x Genomics introduced its own imaging-based ST platform, Xenium, which leverages ISS alongside NGS to spatially resolve RNA transcripts at the single-cell and subcellular levels. Additionally, 10x Genomics acquired Cartana technology and integrated it into the Xenium platform. Xenium can detect up to 5,000 genes in cells and tissues with spatial context at high resolution (31). One limitation of Xenium compared to CosMx SMI is the lack of protein detection; however, 10x Genomics has stated that this feature is in development and will be available in the future. Xenium is a relatively new platform, and peer-reviewed studies using this technology in CRC research have not yet been published. Nevertheless, a CRC Xenium dataset is available for analysis on the 10x Genomics website.

Prior to its acquisition by 10x Genomics, Cartana technology was used in two key publications focusing on CRC research. Sallinger et al. addressed the challenge of deciding whether to administer adjuvant chemotherapy for stage II colon cancer patients by investigating the spatial tissue composition of relapsed versus non-relapsed patients. Using ST Cartana technology, the researchers analyzed a panel of 176 genes related to key cancer processes such as apoptosis, proliferation, angiogenesis, and the tumor microenvironment. They identified a tumor gene signature that enabled the spatial classification of neoplastic and non-neoplastic tissue compartments. Additionally, they identified three differentially expressed genes (FGFR2, MMP11, and OTOP2) in the neoplastic tissue compartments, offering potential biomarkers for predicting relapse and guiding treatment decisions (32).

Andersson et al. aimed to understand the fibrotic rim formed in the desmoplastic histopathologic growth pattern (DHGP) of colorectal cancer liver metastasis (CRLM) using Cartana’s ISS technology. By analyzing 10 chemo-naïve liver metastasis samples, the researchers uncovered molecular and cellular diversity within the rim, confirming the presence of the ductular reaction and cancer-associated fibroblasts (CAFs). These findings offer insights into the origin of the fibrotic rim and propose potential targeted therapies to improve patient survival (33).

### 2.4 Advantages and limitations of imaging-based ST platforms in CRC research

Imaging-based ST platforms offer several advantages for studying gene expression in CRC tumor samples while maintaining spatial context. These platforms provide single-cell and subcellular resolution for the direct visualization of RNA transcripts and tumor architecture, offering novel insights into the spatial organization of gene expression and allowing for the characterization of the TME and cell-to-cell interactions. The CosMx platform provides further insights through its simultaneous protein detection, enabling a more integrated analysis of transcriptomics and proteomics. These platforms are particularly well-suited for targeted spatial transcriptomic analysis once genes of interest have been identified, providing a robust platform for detailed analysis of specific genes.

Despite their advantages, these imaging-based ST methods also face several challenges. MERFISH and other similar techniques require complex, expensive, and labor-intensive protocols due to multiple rounds of hybridization and imaging, which limit throughput and sensitivity. Moreover, the limited tissue size and the need for extensive sample profiling routines make these methods further time-consuming and labor-intensive. For example, while CosMx provides a high level of multiplexing for both RNA and protein detection, the cost and scalability of such high-throughput approaches are often prohibitive for routine clinical application. Other challenges include increasing errors and signal ratios with multiple rounds of hybridization, and optical crowding. Future advancements in automation, cost reduction, and scalability will be essential for wider adoption of these technologies in CRC research and personalized medicine.

## 3 NGS-based spatial transcriptomics in colorectal cancer research

**Figure 2:**
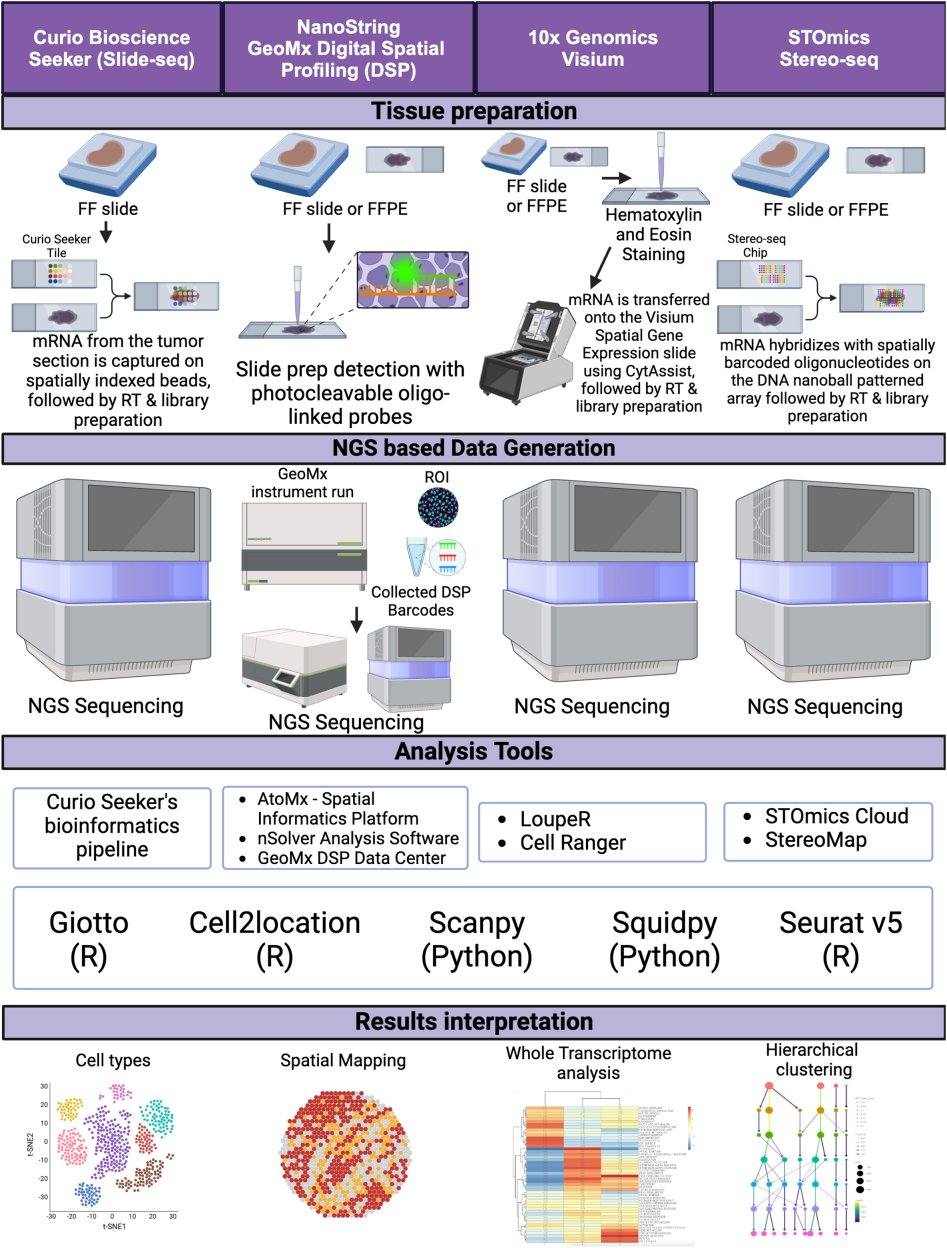
NGS sequence-based spatial transcriptomics. A schematic diagram depicting the workflow for NGS sequence-based spatial transcriptomics platforms, from tissue preparation to data generation, including analysis tools and data interpretation. FF, fresh frozen; FFPE, formalin-fixed paraffin-embedded, RT, reverse transcription, ROI, region of interest. Created in BioRender. Carranza, F. (2024) BioRender.com/e43k544.

Next-generation sequencing (NGS)-based spatial transcriptomics (ST) technologies have significantly advanced our understanding of the spatial organization of gene expression within tissues. By capturing spatial context in tissue samples, these platforms enable the mapping of molecular landscapes, offering high-resolution insights into cellular heterogeneity and cell-cell interactions. Such capabilities are particularly valuable in studying the tumor microenvironment (TME) of CRC, where tumor heterogeneity plays a critical role in disease progression and therapy resistance (**Figure 2**).

### 3.1 Spatial transcriptomics and high-definition spatial transcriptomics

The earliest use of NGS technology for spatial context in tissue samples was demonstrated by Stahl et al. in 2016 using a technique called “Spatial Transcriptomics” (34). This method involved annealing fixed tissue samples directly onto an array with wells spaced 100 μm apart, each containing spatially barcoded reverse transcription primers. These primers capture RNA transcripts for subsequent RNA sequencing, allowing the measurement of gene expression across large regions of tissue sections. However, this method’s resolution was limited to the spatial distribution of groups of cells (3–30 cells per well), which restricted its ability to capture gene expression at the single-cell level (34).

To overcome this limitation, the Stahl group introduced the “High-Definition Spatial Transcriptomics” (HDST) methodology, which uses wells spaced 2 μm apart. This advancement allows for subcellular resolution, capturing gene expression data at a higher resolution and facilitating the study of both intercellular and intracellular interactions (35). HDST provides the foundational technology for 10x Genomics’ NGS ST platforms, including Visium and Visium HD, which have become pivotal in CRC research.

### 3.2 Slide-seq and slide-seqV2

Slide-seq, a subsequent iteration of the Spatial Transcriptomics method, was developed to achieve near single-cell resolution (36). This ST method has since been commercialized under Curio Biosciences Seeker platform. This method involves randomly distributing spatially and DNA-barcoded beads on a slide with beads spaced 10 μm apart. By reducing the bead size and increasing the density, Slide-seq provides finer spatial mapping of gene expression than the original Spatial Transcriptomics. The introduction of Slide-seqV2 further enhanced this method’s efficiency by an order of magnitude (37). Currently, one limitation of Curio’s Seeker platform is its inability to process formalin-fixed paraffin-embedded (FFPE) samples. However, they have announced plans to modify the technology to address this.

In CRC research, Slide-seq has been employed alongside Slide-DNA-seq, a related technology that captures genomic mutations. Together, these methods enable high-resolution, multi-omic characterization of intratumoral heterogeneity. For example, Zhao et al. used Slide-seq to investigate whether genetically distinct CRC tumor clones also have distinct spatial transcriptomes, revealing that different clones exhibit unique gene expression profiles associated with tumor progression and immune infiltration (38). This capability demonstrates the potential of Slide-seq combined with Slide-DNA-seq for spatially characterizing tumor evolution and cell states in CRC.

### 3.3 10x genomics visium and visium HD

Building on the advancements of HDST, 10x Genomics released the Visium platform in 2020 and its higher-resolution counterpart, Visium HD, in 2024. The Visium platform uses a hexagonal array with a capture spot size of 55 μm, enabling the capture of gene expression from 1 to 10 cells per spot (24). Visium HD further improves resolution with 2 × 2 μm barcoded squares with no gaps between squares, allowing for single-cell and subcellular resolution (24). This technology provides a comprehensive view of cell, tumor, and tissue architecture and transcriptomics, enabling the visualization of how a cell’s transcriptome affects cell-to-cell interactions within their native tissue environment (23).

Visium has become one of the most frequently utilized ST platforms in CRC research, with over 34 studies employing this technology to date. For instance, Wu et al. used Visium ST combined with single-cell RNA sequencing (scRNA-seq) to create an immune microenvironment atlas of colorectal liver metastasis (CRLM), revealing a high concentration of immune-suppressive M2-like macrophages in CRC metastatic tumors (39). Similarly, Peng et al. explored cancer-associated fibroblasts (CAFs) in the CRC TME, identifying distinct CAF subtypes associated with poor prognosis and tumor progression (40). Liu H et al. investigated the local invasion process, the initial step in metastasis and leading cause of death in CRC, by using Visium ST to analyze four colorectal cancer tissue samples. By comparing pre-cancerous regions, cancer centers, and invasive margins, researchers identified 13 genes were overexpressed in invasive clusters, including CSTB and TM4SF1 correlating with poor progression-free survival (41). Using Visium ST alongside a cell line-derived xenograft (CDX) model, Martinez-Marin et al. explores how the loss of Helicase-like transcription factor (HLTF), a tumor suppressor, leads to metabolic reprogramming within specific regions of the TME, particularly in lymphatic intravascular metastatic niches (42). These studies highlight Visium’s power in uncovering molecular and cellular dynamics in CRC, advancing precision medicine strategies.

### 3.4 Integrating multi-omic approaches with Visium ST

The Visium platform’s versatility is further showcased when integrated with multi-omic approaches, such as bulk RNA-seq, proteomics, and scRNA-seq. Studies like those conducted by Liu et al. and Yuan et al. have leveraged Visium ST to identify key molecular features of CRC, such as endoplasmic reticulum (ER) stress-related gene patterns and tumor heterogeneity markers, to understand tumor biology better and develop targeted therapies (43, 44). Similarly, Feng et al. integrated CRISPR screening with Visium ST data to identify potential therapeutic targets for immune checkpoint inhibitors (ICIs), underscoring the platform’s utility in immuno-oncology research (45).

### 3.5 NanoString GeoMx digital spatial profiling

NanoString’s GeoMx Digital Spatial Profiler (DSP) is another prominent NGS-based ST platform utilized in CRC research. The DSP platform combines spatial gene expression analysis with high multiplexing capabilities, providing subcellular resolution of 10 μm (46). Unlike most ST platforms, DSP is non-destructive to tissue samples, enabling researchers to perform subsequent analyses on the same sample. The technology employs oligo-labeled probes or antibodies linked to unique DSP barcodes via UV-cleavable linkers, which are hybridized to mRNA targets on a tissue slide. After staining with fluorescent antibodies, regions of interest (ROIs) are selected for barcode release, followed by sequencing to quantify gene or protein expression within a spatially defined context (47).

DSP has been applied in multiple CRC studies to investigate gene expression patterns and molecular interactions. For example, Galeano et al. utilized Visium ST and DSP to map intratumoral microbiota interactions in CRC, revealing how microbial niches influence immune and epithelial cell functions in the TME (48). Tanjak et al. performed a DSP analysis of TME regions within KRASmut CMS3 tissues revealed up regulation of specific genes, CD40, CTLA4, ARG1, STAT3, IDO, CD274, that may contribute to immune suppression in the TME. Lastly, Pennel et al. revealed higher expression of checkpoint and hypoxia-associated genes in tumor regions (pan-cytokeratin positive) and reduced lymphocyte receptor signaling in the TME (pan-cytokeratin-/αSMA-) and αSMA-positive areas (49). Such applications illustrate DSP’s potential for providing detailed insights into the spatial molecular landscape of CRC and facilitating translational research.

### 3.6 STOmics stereo-seq

Spatial enhanced resolution omics sequencing (Stereo-seq) utilizes a patterned array of DNA nanoballs (DNBs) generated through rolling circle amplification to create spatially resolved barcode arrays on a chip surface (50). Each DNB contains a unique spatial barcode that, when combined with next-generation sequencing (NGS), allows for the identification of gene expression profiles at subcellular resolution, reaching up to 220 nm (50). This enables highly detailed spatial mapping of RNA transcripts within tissue samples, surpassing the resolution of earlier ST methods like Visium by 10x Genomics and Slide-seq.

In the context of CRC, Stereo-seq’s ultra-high resolution facilitates an unprecedented level of detail in analyzing tumor architecture, cellular interactions, and the TME. By capturing spatial transcriptomics data at a single-cell or even subcellular level, researchers can dissect the complex cellular and molecular landscapes that govern tumor growth, immune evasion, and metastasis (51). For example, the integration of Stereo-seq with scRNA-seq was used to characterize the spatial distribution of cancer-associated fibroblast (CAF) subtypes in CRC before and after neoadjuvant chemotherapy (NAC). Revealing CAFs distinct roles in modulating the immune microenvironment and promoting tumor progression through pathways such as TGF-β and CXCL12-CXCR4 signaling (51). By identifying specific regions where CAFs interact with immune cells, researchers can better understand how these interactions contribute to immune evasion and resistance to therapies like immune checkpoint inhibitors.

### 3.7 Challenges and opportunities

While NGS-based ST technologies have transformed spatial transcriptomics and multi-omics research, they present certain challenges. These include the high cost of sequencing and high-volume data storage, the need for sophisticated computational tools to analyze complex datasets, and the requirement for specialized equipment and expertise. However, the continued optimization and integration of these platforms with advanced bioinformatics and machine learning models offer exciting opportunities for developing more precise and personalized therapies in CRC and other cancers (42).

Overall, NGS-based spatial transcriptomics has expanded the horizons of cancer research, providing high-resolution spatial data that can elucidate the complexities of tumor biology and the tumor microenvironment. As these technologies continue to evolve, they promise to play an increasingly central role in advancing precision oncology.

## 4 Advanced imaging-based spatial proteomics in CRC

Understanding the tumor microenvironment (TME) in CRC is essential for developing precision therapies and improving patient outcomes. Advanced imaging-based proteomics techniques, such as Multi-Epitope-Ligand Cartography (MELC), Co-Detection by Indexing (CODEX), and the PhenoCycler platform, provide powerful spatial proteomic (SP) tools to dissect the spatial complexity of the TME. These technologies enable the simultaneous visualization and quantification of multiple proteins within a single tissue section, offering a high-resolution map of protein interactions and cellular dynamics that drive CRC progression and treatment resistance.

### 4.1 Multi epitope-ligand cartography in colorectal cancer research

One high-throughput fluorescence microscopy–based method, known as multi-epitope-ligand cartography (MELC), subjects a CRC tissue section to serial rounds of incubation using up to 100 antibody-coupled fluorescent tags, followed by a gentle bleaching step to increase fluorophore detection for each cycle and to map and morphologically position groups of cell types and their connections (52). The cost of performing MELC varies according to the experimental design, allowing the visualization of up to 50 proteins on the same sample. This technique has been reported to profile 20 tissue sections from different tumor types, each with a size of 1.5 cm × 1.5 cm and a thickness of 5 μm, and each stained with 32 antibodies in 32 cycles, consuming a total of 8 hours per slide (53).

MELC’s application in CRC research has been instrumental in mapping the spatial organization of immune cells, stromal cells, and cancer-associated fibroblasts (CAFs) within the TME. It enables the detection of low-abundance proteins, which enhances the understanding of key signaling pathways involved in CRC progression, such as the Wnt/β-catenin and TGF-β pathways. However, MELC is limited by the availability of specific antibodies, the need for a single microscopic medium-to-high-power field, a lengthy sampling time compared to other methods, and the generation of complex output data that require advanced computational analysis (52, 53).

### 4.2 Matrix-assisted laser desorption/ionization mass spectrometric imaging

Matrix-assisted laser desorption/ionization mass spectrometric imaging (MALDI-MSI) is a powerful technique used to spatially resolve, detect, quantify, and map hundreds of biomolecules, including proteins, peptides, lipids, and metabolites. This method works by directing a laser beam at a tissue sample, causing the embedded biomolecules to desorb and ionize. The ionized molecules are then analyzed by mass spectrometry, creating an image that maps the distribution of these biomolecules within the tissue. MALDI MSI is highly sensitive and label-free, avoiding the limitations associated with fluorescence-based methods. An additional advantage is its ability to integrate multi-omics data, providing comprehensive insights into the molecular landscape of the tissue. However, this technique does have limitations, including high costs, potential background noise due to its sensitivity, and the need for specialized equipment and bioinformatic expertise for data analysis (54, 55).

In cancer research, MALDI-MSI has primarily been used to study metabolomics within the TME following treatment. However, our literature review identified a study where MALDI-MSI was combined with liquid chromatography-tandem mass spectrometry (LC-MS/MS) to examine the spatial proteomic landscape in colorectal cancer (CRC) liver metastases. This study analyzed peptide abundance across three comparisons: tumor versus stroma, male versus female, and among three patient groups categorized by overall survival (0–3 years, 4–6 years, and 7+ years). A total of 471 peptides were identified, and survival analysis revealed that three peptides (Histone H4, Hemoglobin subunit alpha, and Inosine-5’-monophosphate dehydrogenase 2) were significantly elevated in patients with shorter survival. Additionally, several protein biomarkers were identified as potential targets for drug development (56).

### 4.3 Multiplexed ion beam imaging

Multiplexed Ion Beam Imaging (MIBI) is a platform commercialized by Ionpath through their MIBIscope 3.0. Originally used for imaging human breast tumors by Dr. Michael Angelo and his team, MIBI is a mass spectrometry-based imaging technology that incorporates mass spectral imaging and secondary ion mass spectrometry. This enables the detection of up to 40 protein biomarkers at subcellular resolution. The technology uses metal-labeled antibody cocktails for tissue staining, followed by imaging with a primary ion beam in a time-of-flight (TOF) mass spectrometer. The imaging process is followed by bioinformatic analysis. One advantage of MIBI is that it avoids signal fading, spectral overlap, and autofluorescence since it does not use fluorophores (57, 58).

MIBI has been utilized in colorectal cancer (CRC) research to examine the metabolic characteristics of cell subsets within tumors. Researchers using this technique have uncovered the spatial organization of metabolically repressed cytotoxic T cells and identified the exclusion of clinically relevant CD8+ T cell subsets from the tumor–immune boundary (59). However, MIBI is limited in the number of markers that can measured simultaneously is limited to existing heavy metals, high cost, high level training required and low throughput speed (60).

### 4.4 Imaging mass cytometry

The Hyperion XTI imaging system from Standard Biotools, formerly Fluidigm, is a multiplexed tissue imaging platform that utilizes Imaging Mass Cytometry (IMC). Initially introduced by Giesen et al., IMC combines immunohistochemical and immunocytochemical techniques with laser ablation and CyTOF (Cytometry by Time of Flight) mass cytometry to achieve subcellular resolution for analyzing over 40 protein or RNA biomarkers in tissue samples. This is accomplished using a strategy similar to that of MIBI, employing metal-tagged antibodies instead of fluorescent labels for tissue staining. Imaging is performed through laser ablation, followed by detection using a mass cytometer and TOF mass spectrometer. The platform shares the same advantages as MIBI, including avoiding signal fading, spectral overlap, and autofluorescence. Additionally, it can process up to 40 samples per run. However, IMC also has certain limitations similar to those of MIBI (60–62).

IMC has been employed to characterize the tumor microenvironment (TME) and uncover the spatial interactions between tumor and immune cell populations in CRC tissue. This has provided a framework for analyzing both intra- and intercellular signaling within the TME, which can ultimately guide precision treatment strategies for CRC patients (63). IMC has also been applied in clinical CRC research to spatially profile tumors before and after pembrolizumab treatment. This analysis revealed that granulocytic cells and cytotoxic T cells were significantly closer in proximity in patients experiencing progressive disease compared to those without progressive events (64).

### 4.5 Co-detection by indexing

Co-Detection by Indexing (CODEX), available via Akoya Biosciences’ PhenoCycler-Fusion platform, relies on the use of a multiplex panel of antibodies conjugated to unique oligonucleotide barcodes specific to three or fewer oligonucleotide reporters linked to fluorophores (65). The hybridization process occurs cyclically, so that each new cycle begins right after the washing step responsible for erasing the remaining signals generated in the previous round. Images are obtained in subcellular layers until all protein targets have been detected (66).

In CRC research, CODEX has been applied to characterize the spatial heterogeneity of immune cells and their interactions with tumor cells. For example, CODEX has been used to map the localization of T cell subsets, macrophages, and dendritic cells in different CRC regions, such as the invasive front and the tumor core. This spatial resolution provides critical insights into the organization of immune niches that contribute to immune evasion and metastasis. Furthermore, CODEX data, like other spatial profiling methods, can be integrated with multi-omics data, such as single-cell RNA sequencing (scRNA-seq), to provide a comprehensive view of the TME and identify potential biomarkers for immunotherapy (66, 67).

PhenoCycler-Fusion analyzes one or two slides at a time with an image area of 35 mm × 18 mm, and the cost depends on the experimental design as the panel size and the custom antibody conjugation process vary according to the study. This method allows for rapid, deep, and unbiased spatial phenotyping of complex CRC tissue sections and provides super-resolution imaging offering multiomic profiling as it can analyze both proteins and RNA biomarkers *in situ* within 24 hours. However, a significant limitation of this assay is data management, storage, and interpretation due to the large amount of information generated, and the assay is also limited by the number of biomarkers that can be detected simultaneously (66, 67).

### 4.6 PhenoCycler: a high-throughput platform for spatial biology

Building upon cyclic immunofluorescent imaging, Akoya Biosciences’ PhenoCycler platform employs typical hybridization cycles to accurately detect fluorophores and distribute protein targets at high resolution. PhenoCycler utilizes a photobleaching cocktail to clean out traces of fluorescently tagged antibody staining left by a previous cycle to upgrade the signal-to-noise ratio (68). PhenoCycler slides can contain multiple tissue sections, and the system is capable of processing up to four slides at a time.

PhenoCycler has enabled the mapping of diverse cellular populations and their interactions at high resolution, facilitating a deeper understanding of the TME’s spatial architecture. For instance, PhenoCycler has been used to characterize the localization and interaction of immune cells, such as regulatory T cells (Tregs), tumor-associated macrophages (TAMs), and myeloid-derived suppressor cells (MDSCs), within CRC tissues. This has provided valuable insights into the mechanisms of immune suppression and tumor progression, potentially guiding the development of combination therapies targeting both the immune checkpoint pathways and the TME’s stromal components (69).

However, like other cyclic immunofluorescent imaging platforms, PhenoCycler requires specialized equipment and reagents that may not be affordable for all research settings. Additionally, the technique may generate artifacts or damage the tissue and is not effective in detecting low-abundance or weakly expressed markers (68).

### 4.7 COMET, sequential immunofluorescence

Lunaphore’s COMET platform is a multiplex immunofluorescence-based system that employs sequential ImmunoFluorescence (seqIF) technology, similar technology to Phenocycler. It can perform 40-plex protein panel spatial proteomic experiments and supports applications such as TME profiling, biomarker discovery, validation, treatment evaluation, and clinical use. However, it shares the same limitations as the Phenocycler platform. As a relatively new technology, there are currently no peer-reviewed CRC studies that have utilized COMET in their analyses (70).

### 4.8 MACSima imaging cyclic staining

The MACSima system by Miltenyi is a multiplex immunofluorescence platform that utilizes Miltenyi’s REAfinity recombinant antibodies along with REAdye Lease fluorochrome technology. Although it shares similarities with other multiplex immunofluorescence systems, the MACSima system stands out for its use of photobleaching or fluorochrome release through REAdye Lease fluorochromes following staining and imaging, providing a gentler approach to treating tissue samples. This technology allows the MACSima system to accommodate protein panels with over 120 different antibodies. Nonetheless, the platform encounters challenges such as significant data management and storage demands and limitations due to the available selection of REAfinity recombinant antibodies provided by Miltenyi (71, 72).

In colorectal cancer (CRC) research, MACSima has been utilized to examine human γδ T cell subsets within the spatial context of CRC tumor tissue. This study served as a proof of concept, enabling researchers to explore cell heterogeneity and the tumor microenvironment (TME) to identify clinically relevant characteristics. Another CRC study employing MACSima for spatial proteomic analysis revealed NECTIN2 expression on cancer-associated fibroblasts (CAFs), but not on tumor cells. Additionally, this study correlated NECTIN2 expression with poor prognosis in CRC patients (73, 74).

### 4.9 Applications of spatial proteomics in colorectal cancer

**Figure 3:**
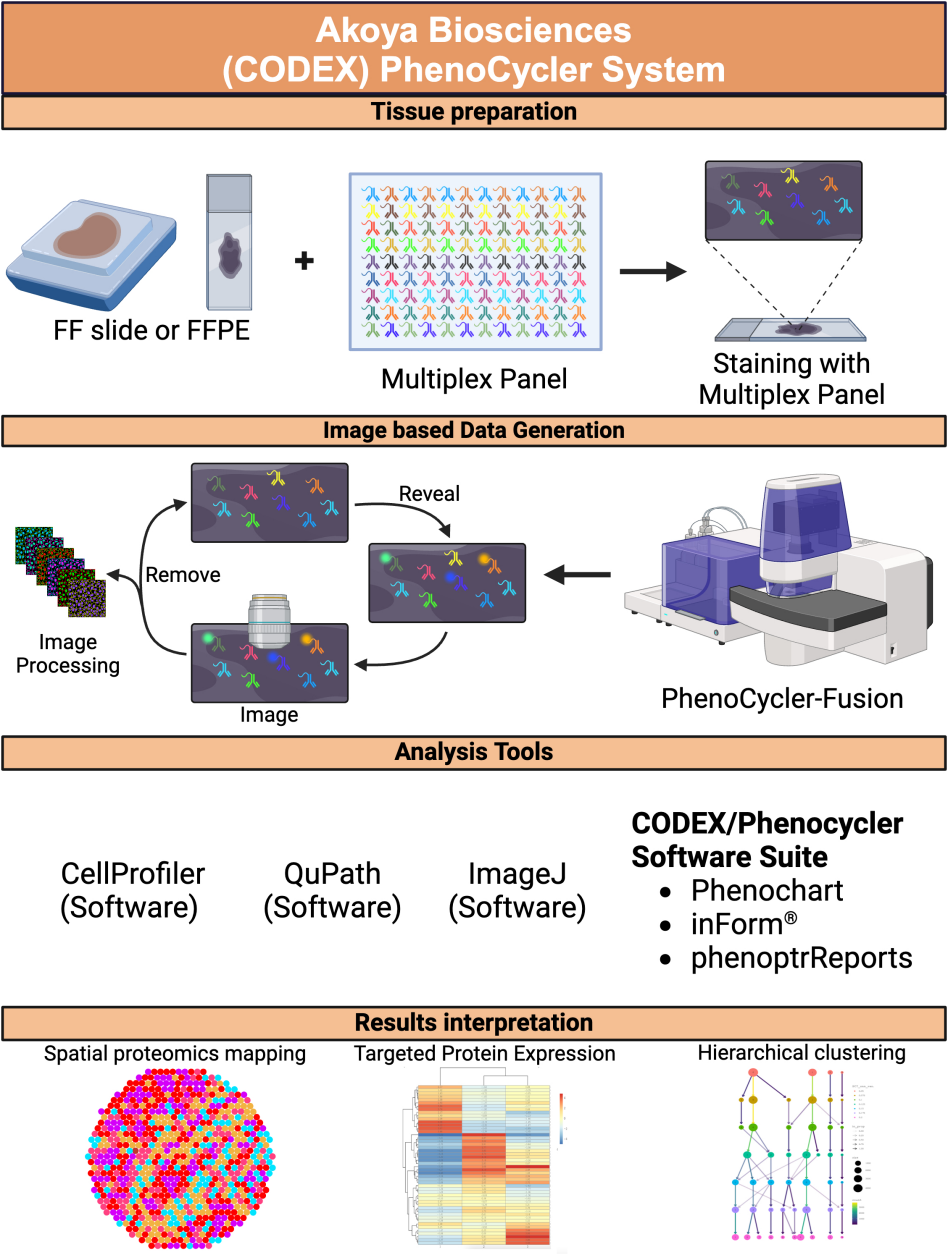
PhenoCycler spatial proteomics. A schematic diagram depicting the workflow for the PhenoCycler spatial proteomics platform, from tissue preparation to image data generation, including analysis tools and data interpretation. FF, fresh frozen; FFPE, formalin-fixed paraffin-embedded. Created in BioRender. Carranza, F. (2024) BioRender.com/r08s204.

The application of spatial proteomics technologies in CRC research has revolutionized our understanding of the tumor microenvironment by providing spatial context to cellular and molecular interactions that drive cancer progression and therapeutic resistance (**Figure 3**).

#### 4.9.1 Mapping Immune Landscapes and Cellular Heterogeneity

These platforms have been particularly effective in revealing the spatial distribution of immune cells and their functional states within CRC tissues. For example, they have been used to identify distinct immune niches characterized by different immune cell compositions and activation states, such as immune-excluded zones rich in fibroblasts and macrophages that contribute to immune evasion (65). Additionally, studies have used these technologies to uncover the roles of various immune cell subsets, such as cytotoxic T lymphocytes (CTLs) and exhausted T cells, in the TME, correlating their presence with patient prognosis and response to immunotherapy (69).

#### 4.9.2 Understanding Stromal and Cancer-Associated Fibroblast Interactions

These platforms have also been pivotal in dissecting the role of stromal cells and CAFs in CRC progression. By mapping the spatial organization of CAFs and their interactions with other cellular components, these techniques have identified pathways such as the TGF-β signaling cascade and CXCL12-CXCR4 axis that promote tumor growth and metastasis (52, 66). Understanding these interactions provides a foundation for developing targeted therapies that disrupt the supportive role of stromal cells in CRC.

#### 4.9.3 Profiling Tumor and Immune Cell Interactions

Using these high-dimensional imaging platforms, researchers have profiled the interactions between tumor cells and immune cells, revealing how the spatial proximity and molecular crosstalk between these cell types influence immune surveillance and escape mechanisms in CRC. For instance, the PhenoCycler platform has been employed to study the spatial dynamics of immune checkpoint proteins, such as PD-1/PD-L1, and their relationship with tumor-infiltrating lymphocytes, thereby identifying potential biomarkers for selecting patients who may benefit from checkpoint inhibitor therapies (67)

#### 4.9.4 Exploring CRC Evolution and Metastasis

By providing a spatial context to genomic and proteomic alterations, these technologies help in understanding the clonal evolution of CRC and the emergence of metastatic subclones. Studies using CODEX and PhenoCycler have revealed the spatial heterogeneity in CRC, where different regions of the same tumor can harbor distinct molecular and cellular characteristics, contributing to treatment resistance and metastasis (75). This information is crucial for developing more effective therapeutic strategies that account for intra-tumor heterogeneity.

#### 4.9.5 Characterizing CRC’s TME using Machine Learning and CODEX

CODEX, enables detailed molecular profiling of cellular environments at subcellular resolution, but identifying disease-relevant microenvironments from these complex datasets remains challenging. A graph neural network leveraging spatial protein profiles was applied to CODEX-imaged tumor specimens, revealing distinctive cellular interactions associated with clinical outcomes in head-and-neck and CRC. This graph-based model, using data from CODEX, was significantly more accurate in predicting patient outcomes than traditional deep learning approaches, providing new insights into the spatial organization of CRC TME that impact prognosis and personalized treatment. Additionally, these graphs coupled with ST may also help in the analysis of disease-relevant motifs (75)

### 4.10 Limitations and future directions

While MELC, CODEX, and PhenoCycler offer powerful tools for spatial proteomics in CRC research, there are several limitations. A major challenge is the need for validated antibodies and probes that are compatible with the cyclic staining and imaging processes. Additionally, these technologies generate vast amounts of data that require advanced computational tools and expertise for accurate analysis and interpretation (66, 68).

Future developments in these platforms may focus on increasing throughput, improving antibody and probe validation processes, and enhancing data analysis capabilities through machine learning and artificial intelligence. Integration with other omics platforms, such as spatial transcriptomics and metabolomics, could provide a more comprehensive understanding of CRC biology at multiple molecular levels, further advancing the field of precision oncology (69).

## 5 Challenges and recommendations in spatial biology platforms for colorectal cancer research

### 5.1 Challenges

**Figure 4:**
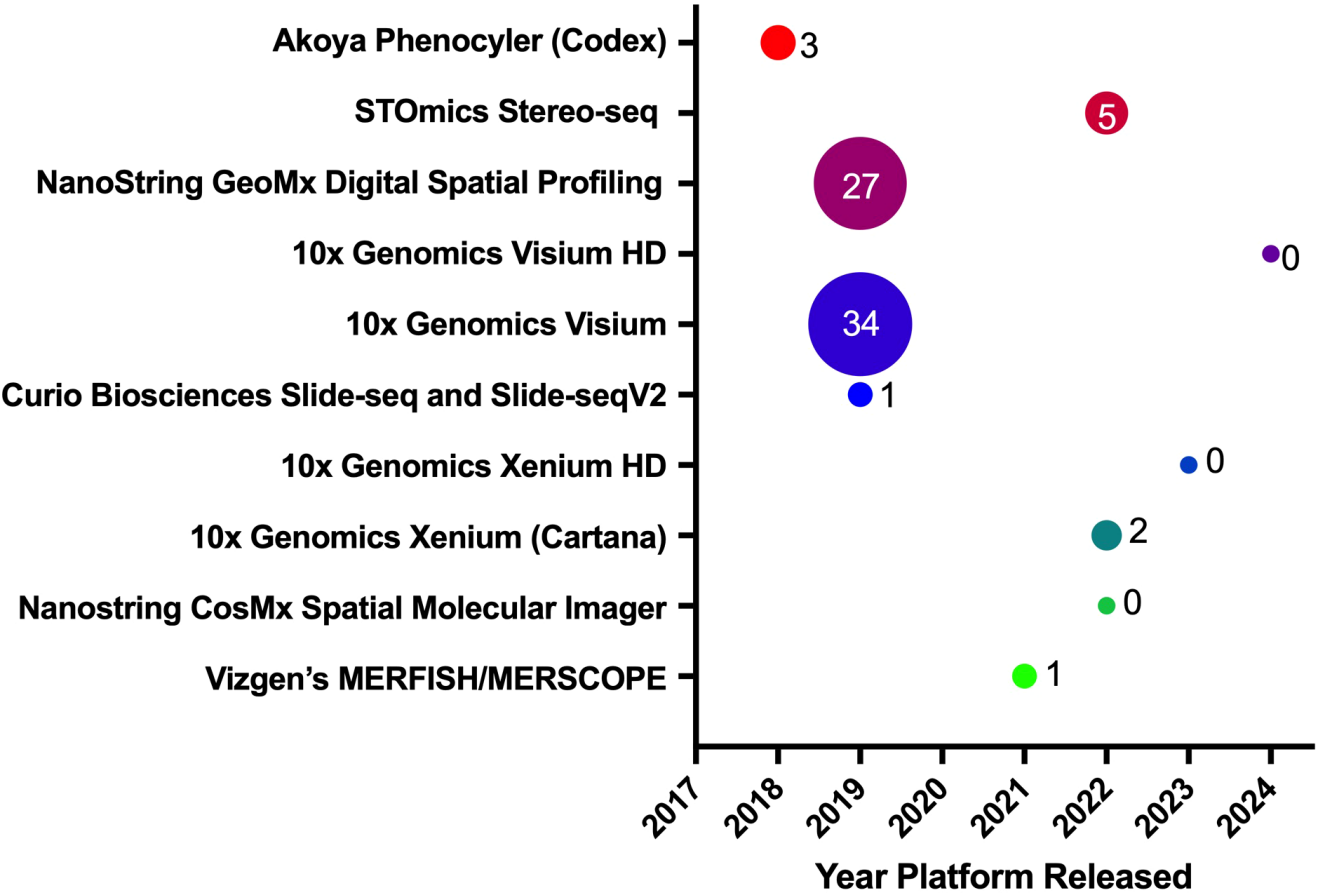
Spatial biology platforms. This panel shows various spatial biology platforms, the year they were introduced to the research market, and their publication impact in CRC. The bubble sizes, along with their associated values, represent the number of publications released using a particular platform, as displayed on the Y-axis.

Despite the transformative potential of ST and SP technologies in CRC research (**Figure 4**), several challenges must be addressed to fully harness their capabilities for clinical and translational applications. These challenges span technical, biological, computational, and practical domains, and overcoming them is crucial for advancing our understanding of CRC and improving patient outcomes.

#### 5.1.1 Technical limitations and scalability

Spatial biology technologies, such as Stereo-seq, CODEX, and Visium HD, offer unparalleled resolution and depth in mapping the tumor microenvironment (TME). However, these technologies often require complex, expensive, and labor-intensive protocols that limit their scalability and routine application in research and clinical settings. The need for specialized equipment, extensive sample preparation, and repeated rounds of imaging or sequencing adds to the complexity and cost of these methods (66, 68). This is particularly challenging for large-scale studies or clinical implementations where high throughput and cost-efficiency are critical. **Table 1** displays the technical requirements and capabilities of all the discussed spatial biology platforms (53, 71, 76–93).

**Table 1:** Spatial biology platforms capabilities.

Spatial Biology Platform	Tissue requirements	Biomolecules captured RNA/Proteins	Resolution	Citations
Imaged Based Spatial Transcriptomics Platforms				
MERSCOPE Ultra	FFPE or fresh frozen sections	1000 + genes (RNA)	Single-Cell	(93)
CosMx SMI	FFPE or fresh frozen sections	6000-plex RNA panel 76-plex protein panel	Single-Cell	(89)
Xenium	FFPE or fresh frozen sections	5000 genes (RNA)	Single-Cell	(83)
NGS Based Spatial Transcriptomics Platforms				
Stereo-seq	FFPE or fresh frozen sections	Whole Transcriptome	Single-Cell	(92)
Curio Seeker (Slide-seq)	Fresh Frozen	Whole Transcriptome	Single-Cell	(77)
Visium	FFPE or fresh frozen sections	Whole Transcriptome	1-10 Cells	(84)
Visium HD	FFPE or fresh frozen sections	Whole Transcriptome	Single-Cell	(84)
GeoMx DSP	FFPE or fresh frozen sections	Whole Transcriptome 20-plex protein panel	20 to 300 Cells	(90)
Spatial Proteomics Platforms				
Multi Epitope-Ligand Cartography (MELC)	FFPE or fresh frozen sections	30-100 proteins	Single-Cell	(53)
MALDIMSI	FFPE or fresh frozen sections	100 + proteins	5-10 Cells	(81, 82, 86, 88, 91)
MIBIScope 3.0	FFPE or fresh frozen sections	40 proteins	Single-Cell	(85)
Hyperion Xti (IMC)	FFPE or fresh frozen sections	40 + proteins	Single-Cell	(80)
PhenoCycler-Fusion	FFPE or fresh frozen sections	100 + proteins	Single-Cell	(78)
COMET	FFPE or fresh frozen sections	40-plex protein panel	Single-Cell	(87)
MACSima	FFPE or fresh frozen sections	100 + proteins	Single-Cell	(71, 79)

This table summarizes the tissue requirements, capabilities, and resolution of the various spatial biology platforms discussed.

#### 5.1.2 Data management and interpretation

The data generated by high-resolution ST platforms is vast and complex, comprising multi-dimensional datasets that capture gene expression at subcellular levels across entire tissue sections. Managing, storing, analyzing, and interpreting these datasets require substantial computational power, sophisticated computational tools, substantial bioinformatics expertise, and advanced statistical methods. The integration of spatial transcriptomic data with other multi-omics data (e.g., proteomics, metabolomics) further complicates the analysis, necessitating more robust pipelines and standardized methodologies for data integration and visualization (66).

#### 5.1.3 Tissue quality and sample preparation

High-quality tissue samples are paramount for successful spatial transcriptomics experiments. Issues such as tissue degradation, RNA quality, and sample handling can significantly affect the results and reproducibility of ST experiments. Additionally, methods like Stereo-seq, which offer ultra-high resolution, require exceptionally well-preserved samples to achieve reliable results. This poses challenges in clinical settings where tissue samples may vary in quality, affecting the feasibility of large-scale studies (50).

#### 5.1.4 Biological complexity and tumor heterogeneity

CRC is characterized by extensive heterogeneity at the genetic, epigenetic, and cellular levels within and between tumors. This heterogeneity complicates the interpretation of spatial transcriptomics data, as the spatial organization and molecular profiles of cells can vary widely across different regions of the same tumor or among different patients. Understanding how these spatial dynamics contribute to tumor progression, immune evasion, and treatment resistance remains a significant challenge (41, 94).

#### 5.1.5 Integration with clinical practice

While spatial biology platforms hold a great promise for enhancing precision oncology, translating these insights into clinical practice is challenging. The high cost, complexity, and need for specialized equipment and expertise make it difficult to implement these technologies in routine clinical workflows. Additionally, there is a need for more standardized protocols, validated biomarkers, and regulatory approvals to ensure that spatial transcriptomic data can be used effectively in clinical decision-making and patient management (65).

### 5.2 Recommendations

To address these challenges, several strategic recommendations can be made to advance the application of spatial biology platforms in CRC research and its integration into precision medicine.

#### 5.2.1 Development of cost-effective and scalable platforms

Future efforts should focus on optimizing ST and SP technologies to reduce costs, increase scalability, and streamline workflows. This could involve the development of automated systems, more efficient protocols, and user-friendly platforms that require less technical expertise. Simplifying the sample preparation process and reducing the number of imaging or sequencing cycles could also enhance the accessibility of these technologies for broader use in research and clinical settings.

#### 5.2.2 Advanced computational tools and data integration pipelines

To manage and interpret the complex datasets generated by ST and SP platforms, there is a need for advanced computational tools that incorporate machine learning and artificial intelligence. These tools should facilitate multi-omics data integration, enabling researchers to correlate spatial transcriptomic data with genomic, epigenomic, proteomic, and metabolomic information. Developing open-source platforms and standardized data processing pipelines will be critical for improving reproducibility and collaborative research efforts.

#### 5.2.3 Improved sample quality and handling protocols

Standardizing protocols for tissue collection, preservation, and handling is essential to ensure the reproducibility and reliability of ST experiments. This includes establishing guidelines for optimal sample preparation, RNA quality control, and storage conditions. Enhancing tissue preservation techniques and using better fixation methods could help maintain RNA integrity, especially for high-resolution platforms like Stereo-seq that require superior sample quality (50).

#### 5.2.4 Targeted approaches to tumor heterogeneity

To address the biological complexity and heterogeneity of CRC, researchers should focus on combining ST and SP data with other technologies such as single-cell RNA sequencing (scRNA-seq) and regular immunofluorescence. This integrative approach can help to deconvolute cellular heterogeneity and identify key spatial patterns associated with tumor progression, immune evasion, and therapy resistance. Developing spatially resolved predictive models and incorporating spatial data into patient stratification strategies could also enhance the clinical relevance of ST research (95, 96).

#### 5.2.5 Translational research and clinical validation

To bridge the gap between research and clinical practice, there is a need for translational studies that validate spatial transcriptomics findings in larger, clinically relevant cohorts. Establishing partnerships between academia, industry, and clinical institutions could facilitate the translation of spatial transcriptomics insights into diagnostic and therapeutic applications. Moreover, developing clinically validated biomarkers and obtaining regulatory approvals for spatially informed tests will be crucial for integrating these technologies into precision oncology workflows.

#### 5.2.6 Enhancing multi-omics integration

Future research should prioritize the development of platforms and protocols that allow seamless integration of spatial transcriptomics with other multi-omics modalities, such as proteomics, epigenomics, and metabolomics. This integrative approach will provide a more comprehensive understanding of CRC biology, revealing novel biomarkers and therapeutic targets. Additionally, enhancing data sharing and collaboration among researchers through centralized repositories and consortia will accelerate discoveries in CRC research.

## 6 Conclusion

While spatial biology platforms have revolutionized CRC research by providing high-resolution insights into tumor biology and the TME, several challenges remain to be addressed to maximize its potential in precision medicine. By developing cost-effective platforms, enhancing computational tools, standardizing protocols, and focusing on translational research, the field can overcome current limitations and advance toward more personalized and effective cancer therapies.
